# Associations between Self-Determined Motivation, Accelerometer-Determined Physical Activity, and Quality of Life in Chinese College Students

**DOI:** 10.3390/ijerph16162941

**Published:** 2019-08-16

**Authors:** Kun Tao, Wenxi Liu, Shanying Xiong, Lodewyk Ken, Nan Zeng, Qingwen Peng, Xiaoni Yan, Junli Wang, Yizhong Wu, Mingzhi Lei, Xianxiong Li, Zan Gao

**Affiliations:** 1 College of Kinesiology and Health Science, Huaihua University, Huaihua 418008, China; 2 School of Kinesiology, University of Minnesota, Twin Cities, MN 55414, USA; 3 Department of Physical Education, Shenzhen Polytechnic University, Shenzhen 518055, China; 4 Department of Kinesiology, Brock University, Ontario L2S 3A1, Canada; 5 Department of Food Science and Human Nutrition, Colorado State University, Fort Collins, CO 80523,USA; 6 School of Physical Education, Hunan Normal University, Changsha 410081, China

**Keywords:** depression, light physical activity, moderate-to-vigorous physical activity, physical function, stress

## Abstract

*Purpose*: To better promote college students’ physical activity (PA) and quality of life (QoL), it is imperative to understand this population’s PA correlates, such as self-determined motivation and perceived competence. However, few studies existed in this area of inquiry among Chinese college students. Thus, the purpose of this study was to examine the relationships among Chinese college students’ self-determined motivation, PA, and QoL. *Method:* A total of 220 college students (115 females; M_age_ = 20.29 years, SD = 2.37; M_BMI_ = 20.67) were recruited from one university in south-central China. Participants were instructed to wear the ActiGraph GT9X Link (ActiGraph, Pensacola, FL, USA) accelerometers for 7 days. A minute-by-minute stepping rate methodology was used to determine participants sedentary behaviors, light physical activity (LPA) and moderate-to-vigorous physical activity (MVPA). Participants’ self-determined motivation (autonomous, controlled, and amotivation), perceived competence, and QoL (physical function, stress, depression, fatigue, sleep, and social issues) were assessed by a battery of validated surveys in June 2017. *Results*: Participants reported moderate–high levels of PA correlates and QoL as the means ranged from 5.5 to 6 (out of 7) for PA correlates and 2.75 to 4 (out of 4) for QoL. The minute-by-minute stepping rate revealed participants had average 580.51 min/day in sedentary, 134.77 min/day in LPA, and 1.57 min/day in MVPA. Regression analyses for physical function, stress, depression, and social issues suggested that the models explained 4%–8% of the variances. Specifically, perceived competence was the negative predictor of the problems with physical function (β = −0.17, *p* < 0.05) and depression (β = −0.18, *p* < 0.01), amotivation was positively associated with depression and stress (*p* < 0.05). Additionally, controlled motivation predicted the ability to participate in social roles and activities (β = 0.22, *p* < 0.05). No significant predictors emerged for fatigue or for sleep. *Conclusions*: Findings suggest Chinese college students’ perceived competence and social support are critical for improving PA and QoL. In addition, strategies are needed to motivate Chinese college students to engage in PA participation and improve overall well-being.

## 1. Introduction

Despite the numerous health benefits of regular physical activity (PA), the prevalence of sedentary and physical inactivity remain high, especially with a significant reduction in PA during the transition from high school to college [1]. According to the World Health Organization (WHO), 23% of adults and 81% of school-going adolescents are not active enough [2]. Previous research has indicated that a lack of motivation appeared to be the major concern for college students participating in consistent PA, and therefore most of them are not regularly active and tend to drop out of structured PA programs [3,4,5,6]. The decreased PA and increased sedentary behavior have been noted among Chinese college students [7]. Motivation influences students’ achievement behaviors and outcomes such as effort, activity choice, and engagement. Self-determination theory (SDT) is a key theory of motivation that has made a substantial contribution to predicting self-determined behaviors [8]. The theory suggests that the degree of self-determined motivation will influence the extent to which an individual engages in and maintain the behaviors. SDT distinguishes between autonomous and controlled forms of motivation. Accordingly, research on PA motivation from the SDT perspective has grown considerably in recent years. The literature had provided substantial evidence for the value of SDT in understanding PA behaviors, and demonstrating the importance of autonomy in promoting sustainable PA [9]. For instance, a previous study examining the role of autonomy and controlled motivation for predicting a large number health behaviors found autonomy was positively related to sustained health-related behaviors (e.g., PA), whereas controlled motivation had fewer effects on the maintenance of those behaviors [10]. To develop effective approaches, it is critical to understand the correlates and determinants that influence Chinese college students’ PA preference and decisions [11,12]. In addition, schools and colleges in China usually pay too much attention to their students’ academic achievements while neglecting students’ well-being and health-related quality of life (QoL) after school.

Numerous studies have suggested that PA improves QoL [13,14,15]. Moreover, research evidence indicated that QoL is a key motivator for PA. In other words, individuals who adopt an active lifestyle and stay in it because they believe PA contributes to their QoL [16,17]. Indeed, this phenomenon aligns well with the self-determination theory. Specifically, as an individual feels PA meets his/her needs and contributes to QoL (e.g., improves physical function, mood, and social relationships), he/she will move up the continuum toward more self-determined motivation [18]. As mentioned earlier, it is important to understand the underlying mechanism of PA behaviors in Chinese young adults to better promote active life and well-being. However, few studies have been conducted in this area to examine relationships of PA correlates and QoL in Chinese college students. Therefore, the present study aimed to examine the relationship between PA correlates and QoL in Chinse college students. In addition, this study used objectively-measured PA to obtain accurate PA outcomes in this population. Study in this inquiry would provide college PA and health professionals information and insights to aid in motivating college students to be more active and improve overall well-being.

### 1.1. Self-Determined Motivation

SDT has been widely used to examine PA behaviors and its correlates in a broad range of settings and populations in the past two decades [18]. SDT uses the concept of innate psychological needs as the basis for integrating the differentiation of goal contents and regulatory processes and the predictions that resulted from those differentiations. According to SDT, a key component in goal pursuit and attainment is the degree to which people are able to satisfy their basic psychological needs as they pursue and obtain their valued outcomes [8]. The basic concept of self-determined motivation is that an individual’s levels of self-determination generally range from autonomous (the most self-determined), controlled, and amotivation (the least self-determined). Autonomous motivation reflects an individual’s intrinsic motivation (i.e., volition or enjoyment) regarding the value of the activity. In contrast, controlled motivation emphasizes extrinsic motivation wherein individuals experience pressure to think, feel, or behave in particular ways. Amotivation refers to a lack of intention and motivation [18]. The continuous levels of self-determined motivation have been used to study motivational factors that influence people’s activity choices, such as diet and exercise adherence [19,20,21,22,23]. Thus far, considerable studies have reinforced the importance of autonomous forms of motivation for promoting sustained PA behaviors over the long term [24,25,26]. Accordingly, the meta-analysis examining associations between motivation and PA indicated autonomous forms of motivation were more strongly and positively associated with PA than controlled motivation [25]. In other words, individuals are more likely to engage in sustained PA and maintain PA behaviors in the long term when they had more internal motives for doing so. In addition, controlled motivation is less likely to be behaviorally adaptive because the behaviors are perceived as inconsistent psychological needs. The controlled motivation may take less of a role than autonomous for promoting sustained PA.

### 1.2. Perceived Competence

In general, perceived competence refers to an individual’s self-perception in their capabilities and ability to control their environment and situation [27]. In sports, it refers to how skilled and effective individuals perceive themselves to do a certain type of PA. Perceived competence is closely related to self-efficacy, which is defined as an individual’s beliefs in one’s own ability to succeed in particular situations [28]. Interventions that focus on promoting self-efficacy may be more effective in the promotion of PA than those do not take motivational factors into consideration [29]. Specifically, self-perceived competence takes an important role in the promotion of PA since physical self-efficacy is an important construct of self-esteem and PA [30]. Previous studies have indicated that children and adolescents with higher self-perceived competence have demonstrated higher levels of leisure-time PA [31]. The study on perceived competence and PA has rarely been studied among college students in China. One recent study, conducted among the U.S. patients with cardiovascular disease, indicated that perceived competence predicted health behavior and health-related QoL [32]. More study is warranted to further examine the associations between perceived competence and QoL in Chinese college-aged adults.

### 1.3. Quality of Life

QoL is a comprehensive concept which evaluates both positive and negative aspects of an individual’s life from a varied perspective. According to WHO, QoL is referred to as a measure of an individual’s physical health, physical, social, and psychological functioning, and a measure of well-being [33]. The QoL was assessed via the validated Patient-Reported Outcomes Measurement Information System (PROMIS), which is a set of person-centered measures that evaluates and monitors physical, mental, and social health in adults and children (The U.S. Department of Health and Human Services). Previous studies have provided evidence that the scale was reliable for assessing health-related outcomes in the U.S. general population and clinical groups [34]. In general, the PROMIS measure of QoL includes assessment of problems with physical function, psychological stress experience, depression, fatigue, sleep disturbance, and the ability to participate in social roles and activities (PROMIS^®^). For PA and QoL, numerous studies have concluded that PA may improve the QoL [35]. Moreover, previous studies have indicated that PA may be a key motivator of QoL, which indicates that individuals engage PA and stick to it when the activities they did contributes to their QoL [18]. However, such relations across the different groups remain largely unexplored. More studies are warranted to consolidate the evidence.

The purpose of this study, therefore, was to examine the relationships in the Chinese college students’ self-determined motivation, objectively-measured PA and QoL, with concern to gender differences. The present study findings may provide information and insights for future researchers and educators who endeavor to promote PA and well-being in college students.

## 2. Methods

### 2.1. Participants

The participants of the present study were recruited from one university in south-central China. The study participants inclusion criteria included: (1) Between 18–25 years old, (2) current registered student, (3) having no self-reported or diagnosed physical or mental disability, and (4) informed consent to participate in the study.

### 2.2. Measurements

#### 2.2.1. Demographic and Anthropometric Information

Participants’ age and gender were obtained from a self-reported demographic questionnaire. Height was measured to the nearest 0.5 cm using a Seca stadiometer (Seca, Hamburg, Germany). Weight and body fat percentage were assessed via the InBody 230 Body Composition Analyzer (Biospace, Seoul, Korea). The body mass index (BMI) was calculated by the weight divided by the square of height (kg/m^2^).

#### 2.2.2. PA

The ActiGraph Link GT9X (ActiGraph, Pensacola, FL, USA) accelerometers were used to capture participants’ PA levels, with daily light PA (LPA), moderate-to-vigorous PA (MVPA), sedentary behavior (SB), and steps per day as the outcomes. While hip-mounted ActiGraph accelerometers have been validated in adults [35], no valid cut points for wrist-worn accelerometers were available to date. Therefore, we used the minute-by-minute stepping rate to calculate PA intensity—a methodology previously employed in other studies of free-living PA to determine SB, LPA, and MVPA duration [36,37].

Congruent with previous studies’ methodology, the following cut points in steps per minute were used: SB: 0–19; LPA: 20–99; MVPA: ≥100. A 60 s epoch was used to facilitate the assessment of minute-by-minute stepping rate [38]. Participants wore the accelerometer for 7 days (the PA data collection should include at least 2 weekdays and 1 weekend day) as suggested from the field-based accelerometer study [39]. To obtain valid data, participants’ PA data must meet with at least 12 h per day of validated wearing time on 2 weekdays and 1 weekend day, processed by ActiLife software (Version 6.13.3; ActiGraph, Pensacola, FL, USA) [39,40]. These data were then exported and analyzed to Microsoft Excel (Microsoft Inc.; Redmond, WA, USA) where “COUNTIFS” and “AVERAGE” functions were employed to determine mean SB, LPA, and MVPA duration in addition to steps per day.

#### 2.2.3. Self-Determined Motivation

Self-determined motivation included the autonomous, controlled motivation, and amotivation (unmotivated) scales from the 15-item self-reported Treatment Self-Regulation Questionnaire (TSRQ). Levesque et al. had examined the validity of TSRQ across settings and health behaviors and found reasonable internal consistency (α = 0.73) [41]. This scale assessed the extent of a person’s motivation for a particular behavior or set of behaviors as relatively autonomous, controlled, or amotivated [41]. A 7-point Likert scale, ranging from “1 = strongly disagree” to “7 = strongly agree” was used for all responses. Sample items were: “The reason I would exercise regularly is because I feel that I want to take responsibility for my own health” (autonomous motivation); “The reason I would exercise regularly is because I would feel guilty or ashamed of myself if I did not exercise regularly” (controlled motivation); and, “I really don’t think about it” (amotivation). Typically, the responses on the autonomous items were averaged to form the reflection of autonomous motivation for the target behavior, the responses on the controlled items were averaged to form the reflection of controlled motivation for the target behavior, and the amotivated responses were also averaged to form the reflection of amotivation.

#### 2.2.4. Perceived Competence

The 4-item Perceived Competence Scale (PCS) reflects an individual’s feelings regarding behaving in healthy ways; that is, the degree that participants feel confident about being to make or maintain a change toward a healthy behavior [42]. Previous studies reported the alpha reliability of the perceived competence items was 0.90 [43]. It is theoretically important to differentiate perceived autonomy (assessed with the TSRQ) from perceived competence (assessed with the PCS). The healthy behavior assessed in this study was regular participation in PA. The measure uses a 7-point Likert response scale ranging from “1 = strongly disagree” to “7 = strongly agree” and a sample item is: “I feel confident in my ability to exercise regularly”. The mean scores were calculated to form the reflection of perceived competence for regular participation in PA.

Notably, as the self-reported measures used in the present study were developed based on western culture, therefore, the validation of the instruments for Chinese college students was conducted prior to data analysis. It is essential to examine the cross-cultural validity of instruments for respondents with different cultural backgrounds [44]. Previous literature has suggested that translating the instruments by a team with expertise in multi-cultural and multi-lingual contexts were able to remedy the bias [45]. Besides, an independent back-translation was adopted to detect the survey items bias. In the present study, the surveys were translated by the researchers who have expertise in both Chinese and English. The translations were evaluated by three bilingual (Chinese/English) professionals. Then, the surveys were translated back to English. The bilingual professionals conducted a comparison of the original and back-translated English version, and no deviations in meaning were found.

#### 2.2.5. Quality of Life

The 24-item Patient-Reported Outcomes Measurement Information System (PROMIS) was used to assess participants’ physical, mental, and social well-being [46]. One recent validation study has found the good reliability and validity of Chinese version PROMIS for measuring symptoms and function among children and adolescents in China [47]. The PROMIS scale assesses participants’ physical function, anxiety, depression, fatigue, sleep disturbance, and the ability to participate in social roles and activities. Each subscale housed 4-items and used a 5-point Likert response scale. For physical function, the response scale ranged from “without any difficulty” (1) to “unable to do” (5) and a sample item was: “Are you able to do chores such as vacuuming or yard work?”. For the anxiety and depression sub-scales, the response scale ranged “never” (1) to “always” (5) and a sample item was: “In the past 7 days, I found it hard to focus on anything other than my anxiety.” The response scale for the fatigue, sleep disturbance, and ability to participate in social roles and activities sub-scales ranged from (1) “not at all” to (5) “very much” and sample items were “During the past 7 days, I feel fatigued” and “In the past 7 days, I had a problem with my sleep”. The mean scores were calculated as study outcomes.

### 2.3. Procedures

The procedure was approved by the ethics committee at the University (no.: 20170606P1) and consent was obtained from participants prior to the start of this study. All data were collected by the primary researchers during regularly scheduled college physical education classes in June 2017. Participation was voluntary, and no extra credit was awarded to participating students. Participants were asked to wear the ActiGraph Link accelerometers on the wrist for 7 days. Accelerometers were required to be worn all the time during except bathing and swimming. The motivation, perceived competence, and QoL survey were collected during physical education classes.

### 2.4. Data Analysis

All analyses were performed using IBM-SPSS 25.0 (IBM Inc., Armonk, NY, USA). Data were first screened for the outliers and normality of distributions. Outliers were adjusted to lessen the impact of extreme scores. Specifically, if a score on outcome measures was 3 median absolute deviations (MAD) away from the median of the residuals, then that value was classified as an outlier and was truncated. Of the 7 variables included in the current study, 3 continuous variables had at least one missing value, and across the entire dataset, 0.08% of the values were missing. All the missing values were replaced using multiple imputations.

Prior to data analyses, the validity of data collected from participants was examined. To obtain valid PA data, participants’ PA data must meet with at least 12 h per day of validated wearing time on 2 weekdays and 1 weekend day. After the initial examination, a total of 220 participants had valid data for further analyses. Descriptive statistics were thereafter used to describe the participants’ demographic variables, daily duration of step per minute on SB, LPA, MVPA, step per day, self-determined motivation (autonomous, controlled motivation, and amotivation), perceived competence, and QoL indices (problem with physical function, anxiety, depression, fatigue, sleep disturbance, and ability to participate in social roles and activities). Second, Pearson product-moment correlations were computed to examine the interrelationships among the study variables. Third, multiple regression was used to explore the predictive relationships between self-determined motivation, perceived competence, and the six QoL subscales. Fourth, the multivariate analysis of variance (MANOVA) was used to examine the gender difference regarding all study outcomes. The statistical significance was set at 0.05.

Ethics Approval and Consent to Participate: The procedures were approved by the ethics review committees at the participating Universities and consent was obtained from all necessary levels prior to the start of this study.

Consent for Publication: All authors have approved the manuscript as submitted. My co-authors and I are responsible for reported research and do not have any interests that might be interpreted as influencing the research, and AMA ethical standards were followed in the conduct of the study. All authors acknowledge ethical responsibility for the content of the manuscript and will accept the consequences of any ethical violations.

Availability of Data and Material: The data will initially be collected on paper and will subsequently be transferred to electronic media on a computer spreadsheet for export to statistical packages. The paper data will be stored in a locked file cabinet within the principal investigator’s office for a minimum of three years after the last participant has completed data collection. Electronic media files of the data will also be stored for a minimum of three years on the principle investigator’s computer and on a computer within the University of Minnesota’s Physical Activity Epidemiology Laboratory. Both computers are password encrypted and are only capable of being accessed by the principal investigator. The data collected during the current study will not be released or shared with anyone aside from participants.

## 3. Results

Descriptive statistics indicated a total of 220 college students (115 females; Mage = 20.29, SD = 2.37; M_BMI_ = 20.67, SD = 3.12; M_%BF_ = 21.92%, SD = 7.94) were included for data analyses. Table 1 shows the descriptive statistics results of participants’ characteristics and study outcomes. In detail, participants reported average score of 4.80 on autonomous (SD = 1.16; out of 7), 3.32 on controlled motivation (SD = 1.20; out of 7), 2.85 on amotivation (SD = 1.38; out of 7), and 4.33 on perceived competence (SD = 1.26; out of 7). Regarding QoL subscale mean scores (out of 5), participants reported an average of 1.14 on problems with physical function (SD = 0.34), 1.83 on anxiety (SD = 0.77), 1.63 on depression (SD = 0.74), 2.31 on fatigue (SD = 0.88), 2.66 on poor sleep (SD = 0.91), and 1.86 on social problems (SD = 0.71) As shown in Table 2, the correlation analysis indicated study variables had modest-to-strong correlations ranged from −0.14 to 0.63. The minute-by-minute stepping rate revealed participants had an average 580.51 min per day on sedentary behaviors, 134.77 min per day on LPA, 1.57 min per day on MVPA, and accumulated 8105.59 daily steps.

Table 3 shows the predictive associations between self-determined motivation, perceived competence, and QoL. Regression analyses for physical function, anxiety, depression, and social interactions indicated the respective predictors explained by 4%–8% of the variances. Specifically, perceived competence negatively and significantly predicted physical function (β = −0.047, *p* = 0.049); amotivation predicted depression (β = 0.130, *p* = 0.016); and controlled motivation predicted the ability to participate in social roles and activities (β = 0.130, *p* = 0.016). There was no other significant predictive association between the self-determined motivation and the QoL subscales.

Table 4 shows the gender differences across all the outcomes. MANOVA analyses revealed that there were significant gender differences on controlled motivation (F (1, 219) = 8.41, η_p_^2^ = 0.037, *p* = 0.004) and perceived competence (F (1, 219) = 8.36, η_p_^2^ = 0.037, *p* = 0.004). Specifically, males had higher controlled motivation (M = 3.56, SD = 1.18) and perceived competence (M = 4.59, SD = 1.25) compared to females (controlled motivation: M = 3.10, SD = 1.18; perceived competence: M = 4.10, SD = 1.23). There were no gender differences observed in other outcomes.

## 4. Discussion

The present study examined relationships between self-determined motivation (autonomous, controlled motivation, and amotivation), perceived competence, PA, and QoL in Chinese college students. This study adopted an objective measure of PA and validated it by using a minute-by-minute stepping rate methodology. The results indicated that current participants spent most of their awake time in sedentary behaviors (580.51 min/day) and LPA (134.77 min/day). Notably, the current participants did not meet the recommended amount of MVPA, with only 1.57 min per day observed. WHO has suggested that adults aged 18–64 years old should engage in at least 150 min of MVPA throughout the week, and the activity should be performed in bouts of at least 10 min duration [48]. The findings are similar to previous studies that used a minute-by-minute stepping rate to determine 3744 U.S. adults’ free-living stepping cadence patterns, which concluded self-selected walking at 100+ steps per minute was a rare phenomenon in a large free-living sample of U.S. adults, but study participants did accumulate about 30 min per day at a cadence of 60+ steps per min [37]. The current study’s findings echo a recent study which revealed the rising prevalence of low-level PA and high screen time among Chinese college students [49]. It is well known that economic and technological improvements increase screen time among young adults in developing countries. The increased screen time resulted in reduced PA, which may explain current participants spending a large amount of time in sedentary behaviors (about 9.68 h/day) in the present study. Although Chinese universities offered physical education classes for students, the curriculum content and quality varied tremendously. Our data indicated that most of college students’ active time were attributed to the LPA but not MVPA, suggesting that college students may have less interests in the physical education content and format. Indeed, a previous study examined Chinese college students’ expectancy-value motivation in relation to physical education and self-initiated PA and found students had lower perceived value for physical education [50]. Therefore, future studies are needed to examine Chinese college students’ motives for physical education, and educators need to develop effective and enjoyable strategies to promote MVPA during class time.

Regression analysis further revealed a significant negative association between perceived competence and the problem with physical function. In other words, the findings indicated that college students who had higher perceived competence may have less problem with their physical function while performing certain activities. When facing a PA-related task, people who feel confident of their capability in completing the task may have fewer problems with their physical functioning. This finding is consistent with the previous study which evaluated the effect of perceived health competence on participants’ abilities to achieve health-related goals, health behaviors, and health-related QoL, and concluded that perceived competence was positively associated with PA and QoL in adults [32]. The finding implies that college students in this study demonstrated fewer problems with their physical function when they believed that they would do well on certain activities [51]. The implication of this finding provides insights to college health and PA professionals for developing effective programs, for instance, programs should consider fostering students’ perceived competence, and set easy to follow, realistic, and progressive goals, to motivate PA participation and adherence.

Furthermore, our data indicated controlled motivation was positively associated with the ability to participate in social roles and activities. Social factors such as support from family and friends were considered as extrinsic motives to motivate individuals engaging in PA. A previous study has highlighted the importance of positive support from family and friends in increasing social engagement and psychological well-being [52]. The results indicated extrinsic motives such as support from friends and family may facilitate improvement in college students’ abilities to participate in social activities. This finding suggests future studies should consider social support as a means for motivating PA participation and improving QoL among Chinese college students. Notably, we did not observe the significant relationship between autonomous and QoL, however, the controlled motivation seems to play a more important role in improving health-related QoL. This finding is consistent with the previous study, which found the most popular college student motivation was extrinsic motivation rather than intrinsic motivation [53]. Specifically, the previous finding indicated that controlled motivation may have more impact than autonomous motivation for predicting initial or short-term PA behaviors, but intrinsic motivation (autonomous) may be more predictive of long-term PA maintenance. Future studies may consider examining the role of different motives (e.g., health/fitness and body-related activities) in this population for promoting PA and QoL.

Amotivation was positively associated with anxiety and depression in this study. According to SDT, intrinsic motivation and all forms of extrinsic motivation involve different levels of intentionality and motivation, but amotivation stands in contrast to intrinsic and extrinsic motivation, which represents a complete lack of self-determination with respect to the target behavior [8]. A previous study indicated that amotivation was associated with the poorest performance and mental-health outcomes (e.g., anxiety) It is critical to address motivational strategies for motivating college students to be more physically active and to encourage mental well-being. A large amount of evidence has indicated the close link between PA and motivation, therefore, identifying the PA-related motives take an important role in developing effective PA and health-related programs in college students.

Notably, there was no significant predictive association between PA and self-determined motivation, perceived competence, and QoL. One recent meta-analysis study examining self-determined motivation and PA indicated there was substantial heterogeneity in most associations and many studies had methodological shortcomings [25]. Although the surveys were translated by the bilingual researchers and validated by the bilingual experts, the cultural differences might influence participants’ responses. When adopting surveys from western culture, future studies need to develop strategies for improving the compatibility of surveys on Chinese college students.

The results of this study indicated that males had more controlled motivation and perceived competence than females. Controlled motivation reflects engaging activity for externally referenced reasons such as gaining rewards or avoiding punishment. Individuals participating in PA for controlled reasons feel a sense of obligation and pressure when engaging in PA [10]. Our result indicated males perceived more controlled reasons for participating in PA than females. While numerous studies suggested that intrinsic motivation (autonomous) was the key motive for maintaining sustained PA, researchers examining college students’ PA motivation found that the most popular student motivation was extrinsic (controlled motivation) instead of intrinsic motivation [53]. Researchers examining college students’ motivation for PA concerning gender differences found that men had a higher level of motivation than women in activities characterized as being challenging, a competition, having social recognition, and requiring strength and endurance [54]. Therefore, future studies are needed to differentiate the motives between male and female college students in China with the goal of effectively motivating PA participation in this population.

Several limitations of the study must be acknowledged. First, the generalizability of findings from this study needs to be carefully considered, as all participants were from one single university in south-central China. Second, due to the nature of the observational study, causal relationships among the outcome variables in this study cannot be determined. Future study should adopt a longitudinal cohort design to explore the PA and QoL determinants of college students. Third, it is worth noting that the minute-by-minute stepping rate used in the current study only considered the walking steps, but does not address the fact that many human activities go beyond simple ambulation. These include complex movements that may engage the upper body. Such movements are obviously not well captured by a simple rendering of steps taken or cadence. Thus, the other activities that go beyond walking may not be captured, suggesting that participants’ PA levels in our study might be underestimated by this approach. That is, our observations are not comparable to other studies using different PA calculations such as GGIR. Therefore, we encourage future studies to further explore such relationships using other PA assessments. Lastly, although researchers in this study translated English surveys to Chinese and tested the validity of the Chinese version, cautions are warranted when interpreting the results from the present study.

## 5. Conclusions

In conclusion, the present study examined relationships between Chinese college students’ self-determined motivation, perceived competence, PA, and QoL. The findings suggest perceived competence is critical when making PA-related decisions and choice, and is related to improving QoL in Chinese college students. In addition, it is important to develop strategies to motivate Chinese college students to engage in PA. Lack of motivation for participating in PA may increase the risk of depression, thus affecting their QoL. Lastly, support and interactions from friends and family members are important in motivating Chinese college students to engage in PA and improve their abilities to participate in social roles and activities.

### Implications

The present study’s finding suggests that it is critical for educators to foster college students’ perceived competence to motivate Chinese college students to engage in sustainable PA. Strategies for enhancing the motivations of PA participation are needed to promote an active lifestyle among Chinese college students. In addition, future studies need to consider gender differences concerning how to foster perceived competence and motivation for males and females. It is important to note that, when developing PA programs among Chinese college students, we should consider social support and interaction to improve students’ overall well-being.

## Figures and Tables

**Table 1:** Descriptive statistics on study variables.

Variables	Mean	SD
Age (years)	20.29	2.37
BMI	20.67	3.12
BF (%)	21.92	7.94
Autonomous (out of 7)	4.80	1.16
Controlled (out of 7)	3.32	1.20
Amotivation (out of 7)	2.85	1.38
Perceived competence (out of 7)	4.33	1.26
Problem with PF (out of 5)	1.14	0.34
Anxiety (out of 5)	1.83	0.77
Depression (out of 5)	1.63	0.74
Fatigue (out of 5)	2.31	0.88
Poor sleep (out of 5)	2.66	0.91
social problem (out of 5)	1.86	0.71
Sedentary behavior (min/day)	580.51	50.54
LPA (min/day)	134.77	6.13
MVPA (min/day)	1.57	2.33
Steps	8105.59	2188.77

Note: SD, standard deviation; BMI, body mass index; %BF, percent of body fat; PF, physical function; LPA, light physical activity; MVPA, moderate-to-vigorous physical activity.

**Table 2:** Correlations among all the outcome variables.

Variables	1	2	3	4	5	6	7	8	9	10	11	12	13	14
Autonomous	-													
Controlled	0.499 **	-												
Amotivation	−0.105	0.403 **	-											
Perceived Competence	0.626 **	0.379 **	−0.06	-										
Problem with PF	−0.137 *	−0.003	0.046	−0.182 **	-									
Anxiety	0.05	0.184 **	0.194 **	0.021	0.145 *	-								
Depression	0.052	0.157 *	0.192 **	−0.011	0.249 **	0.758 **	-							
Fatigue	0.062	0.113	0.105	0.001	0.078	0.457 **	0.491 **	-						
Poor sleep	−0.033	0.047	0.071	−0.04	0.151 *	0.485 **	0.422 **	0.400 **	-					
Social problem	0.114	0.249 **	0.145 *	0.096	0.145 *	0.424 **	0.515 **	0.352 **	0.274 **	-				
Time in MVPA	0.021	0.055	−0.051	0.065	0.01	0.121	0.066	0.033	0.126	−0.012	-			
Time in LPA	0.023	0.01	−0.048	0.054	0.131	−0.023	0.011	0.02	−0.065	−0.046	0.105	-		
Time in Sedentary	0.014	0.007	0.04	−0.04	−0.150 *	−0.02	−0.016	−0.09	0.056	0.034	−0.103	−0.895 **	-	
Steps per day	−0.006	−0.006	−0.047	0.068	0.122	−0.041	−0.036	−0.016	−0.049	−0.035	0.233 **	0.904 **	−0.823 **	-

Note. MVPA = moderate-to-vigorous physical activity; LPA = light physical activity. ** Correlation is significant at the 0.01 level (2-tailed); * Correlation is significant at the 0.05 level (2-tailed).

**Table 3:** Regression analysis of self-determined motivation and quality of life variables.

Dependent Variables	Independent Variables	β	*p*
Physical Function	Autonomous	−0.026	0.356
	Controlled Motivation	0.033	0.215
	Amotivation	−0.005	0.809
	Perceived Competence	−0.047	0.049
Anxiety	Autonomous	0.014	0.823
	Controlled Motivation	0.083	0.165
	Amotivation	0.079	0.076
	Perceived Competence	−0.020	0.701
Depression	Autonomous	0.047	0.444
	Controlled Motivation	0.063	0.263
	Amotivation	0.087	0.039
	Perceived Competence	−0.056	0.266
Fatigue	Autonomous	0.062	0.406
	Controlled Motivation	0.048	0.483
	Amotivation	0.053	0.302
	Perceived Competence	−0.048	0.429
Sleep Disturbance	Autonomous	−0.029	0.709
	Controlled Motivation	0.049	0.491
	Amotivation	0.026	0.630
	Perceived Competence	−0.028	0.655
Ability to participate in social roles and activities	Autonomous	0.000	0.994
	Controlled Motivation	0.130	0.016
	Amotivation	0.030	0.457
	Perceived Competence	0.009	0.847

Note: β, unstandardized regression coefficient.

**Table 4:** MANOVA analysis results of gender difference on study variables.

Variables	Mean		SD		*p*
	Male	Female	Male	Female	0.004
Controlled Motivation	3.56	3.10	1.18	1.18	0.004
Perceived Competence	4.59	4.10	1.25	1.23	0.004

Note: SD, standard deviation; Sig., significance; significant level at 0.05.
